# Distribution of *papA* and *papG* Variants among *Escherichia coli* Genotypes: Association with Major Extraintestinal Pathogenic Lineages

**DOI:** 10.3390/ijms25126657

**Published:** 2024-06-17

**Authors:** Valentina Fernández-Yáñez, Patricio Suazo, Claudia Hormazábal, Valentina Ibaceta, Mauricio Arenas-Salinas, Roberto M. Vidal, Francisco Silva-Ojeda, Carolina Arellano, Ignacio Muñoz, Felipe Del Canto

**Affiliations:** 1 Departamento de Biología, Facultad de Química y Biología, Universidad de Santiago de Chile, Av. Libertador Bernardo O’Higgins 3363, Santiago 9170022, Chile; valentina.fernandezy@usach.cl (V.F.-Y.);; 2 Programa de Microbiología y Micología, Instituto de Ciencias Biomédicas, Facultad de Medicina, Universidad de Chile, Av. Independencia 1027, Santiago 8380453, Chile; 3 Centro de Bioinformática Simulación y Modelado, Facultad de Ingeniería, Universidad de Talca, Av. Lircay s/n, Talca 3460787, Chile; 4 Instituto Milenio de Inmunología e Inmunoterapia, Facultad de Medicina, Universidad de Chile, Av. Independencia 1027, Santiago 8380453, Chile; 5 Servicio de Laboratorio Clínico, Hospital Clínico Universidad de Chile, Av. Dr. Carlos Lorca Tobar 999, Santiago 8380453, Chile

**Keywords:** P fimbria, *papA*, *papG*, extraintestinal pathogenic *Escherichia coli*

## Abstract

The pyelonephritis-associated fimbria (P fimbria) is one of the most recognized adhesion determinants of extraintestinal pathogenic *Escherichia coli* strains (ExPECs). Twelve variants have been described for the gene encoding the P fimbria major structural subunit PapA and three variants for the gene encoding the adhesin subunit PapG. However, their distribution among the ExPEC diversity has not been comprehensively addressed. A complete landscape of that distribution might be valuable for delineating basic studies about the pathogenicity mechanisms of ExPECs and following up on the evolution of ExPEC lineages, particularly those most epidemiologically relevant. Therefore, we performed a massive descriptive study to detect the *papA* and *papG* variants along different *E. coli* genotypes represented by genomic sequences contained in the NCBI Assembly Refseq database. The most common *papA* variants were F11, F10, F48, F16, F12, and F7-2, which were found in significant association with the most relevant ExPEC genotypes, the phylogroups B2 and D, and the sequence types ST95, ST131, ST127, ST69, ST12, and ST73. On the other hand, the *papGII* variant was by far the most common followed by *papGIII*, and both were also found to have a significant association with common ExPEC genotypes. We noticed the presence of genomes, mainly belonging to the sequence type ST12, harboring two or three *papA* variants and two *papG* variants. Furthermore, the most common *papA* and *papG* variants were also detected in records representing strains isolated from humans and animals such as poultry, bovine, and dogs, supporting previous hypotheses of potential cross-transmission. Finally, we characterized a set of 17 genomes from Chilean uropathogenic *E. coli* strains and found that ST12 and ST73 were the predominant sequence types. Variants F7-1, F7-2, F8, F9, F11, F13, F14, F16, and F48 were detected for *papA*, and *papGII* and *papGIII* variants were detected for *papG*. Significant associations with the sequence types observed in the analysis of genomes contained in the NCBI Assembly Refseq database were also found in this collection in 16 of 19 cases for *papA* variants and 7 of 9 cases for the *papG* variants. This comprehensive characterization might support future basic studies about P fimbria-mediated ExPEC adherence and future typing or epidemiological studies to monitor the evolution of ExPECs producing P fimbria.

## 1. Introduction

Extraintestinal pathogenic *Escherichia coli* strains (ExPECs) are a common cause of morbidity and mortality in humans and other animals [1,2]. The ExPEC group is widely diverse regarding its genotypes and repertoires of virulence factors. It includes several pathotypes capable of causing infections at diverse human body sites, such as uropathogenic *E. coli* (UPEC), sepsis-associated *E. coli* (SEPEC), and neonatal meningitis-associated *E. coli* (NMEC) [3]. In animals, ExPECs can cause diseases such as colibacillosis in chickens and swine and mastitis in cattle, leading to significant economic losses in poultry and the farm industry [4,5,6]. In addition, ExPECs can cause urinary tract infections in cattle and accompaniment pets, such as dogs and cats [7,8,9]. 

It is well accepted that the main reservoir of ExPECs is the gastrointestinal tract of humans and other animals [10], which is also the reservoir of diarrheagenic *E. coli* and the habitat of commensal strains [11,12,13,14]. However, in contrast to other pathotypes or commensal strains, ExPECs can colonize, persist, and disseminate into extraintestinal tissues [3]. Several virulence factors are determinants of this capacity, including adherence structures, toxins, iron-capture systems, and the capsule [3].

The wide diversity of ExPECs regarding their genotypes and virulence factor repertoire has hampered the identification and use of specific molecular markers to recognize ExPECs for diagnostic purposes and also in the context of epidemiological studies [10,11]. Numerous attempts performed over several years have allowed for the identification of some common features at the strains’ genotype level. For example, ExPECs correspond mainly to isolates belonging to *E. coli* phylogroups B2 and D [15]. In addition, in the case of extraintestinal infections in humans, sequence types ST69, ST73, ST95, ST131, and ST393 are the most frequently isolated and have been recognized as pandemic lineages [16]. ST10, ST12, ST14, ST117, ST127, ST141, and ST405 are usually found too, depending on the geographic location [17,18]. 

The pyelonephritis-associated fimbria (P fimbria) is one of the most recognized ExPEC virulence factors [19]. P fimbria is a structure assembled by the chaperone–usher pathway, composed mainly of thousands of copies of the PapA protein, the major structural subunit, and, in minor proportion, the structural subunits PapK, PapE, and PapF and the tip subunit PapG, to which the adhesin activity is attributed [20]. The assembly process is assisted by the periplasmic chaperone PapD and the usher PapC, an outer membrane porin-like protein in which the polymerization of structural subunits and then the fimbria’s exposition to the cell surface occur [20]. Two other proteins, PapH and PapJ, are also part of the system, but their roles have not been fully established. Data suggest that PapH acts as an anchor protein that helps to maintain the fimbria attached to the bacterium, and PapJ would be a second periplasmic chaperone [21,22]. 

The relevance of the P fimbria in ExPEC pathogenicity has been established, mainly for the adherence capacity of UPEC strains, which is why it has been proposed as a potential basis for developing anti-virulence therapies [23]. Diversity in the components of P fimbria is one of the obstacles to overcome to gain insights into its molecular mechanisms and move forward with potential applications. Sequence variability has been reported for PapA and PapG, for which 12 and 3 variants have been established, respectively. Variants F7-1, F7-2, F8, F9, F10, F11, F12, F13, F14, F15, F16, and F48 have been described for PapA [24], while genetic variants *papGI*, *papGII*, and *papGIII*, have been established for *papG* [25]. A few studies have analyzed the presence and distribution of these variants in separated sets of ExPECs, especially during the last years, in which multiple characterizations have been supported using massive genome sequencing technologies [26,27,28]. However, to our knowledge, the distribution of *papA* and *papG* variants among a highly diverse set of *E. coli* genotypes has yet to be addressed. Given the high relevance of the P fimbria in ExPEC pathogenicity, we believe the current landscape of this diversity should be established. Therefore, in this work, we report the analysis of *E. coli* genomes contained in databases to find associations between *papA* and *papG* variants with the phylogroup and sequence types. In addition, we looked for these associations in a set of newly sequenced UPEC strains isolated in Chile. 

## 2. Results

**Figure 1:**
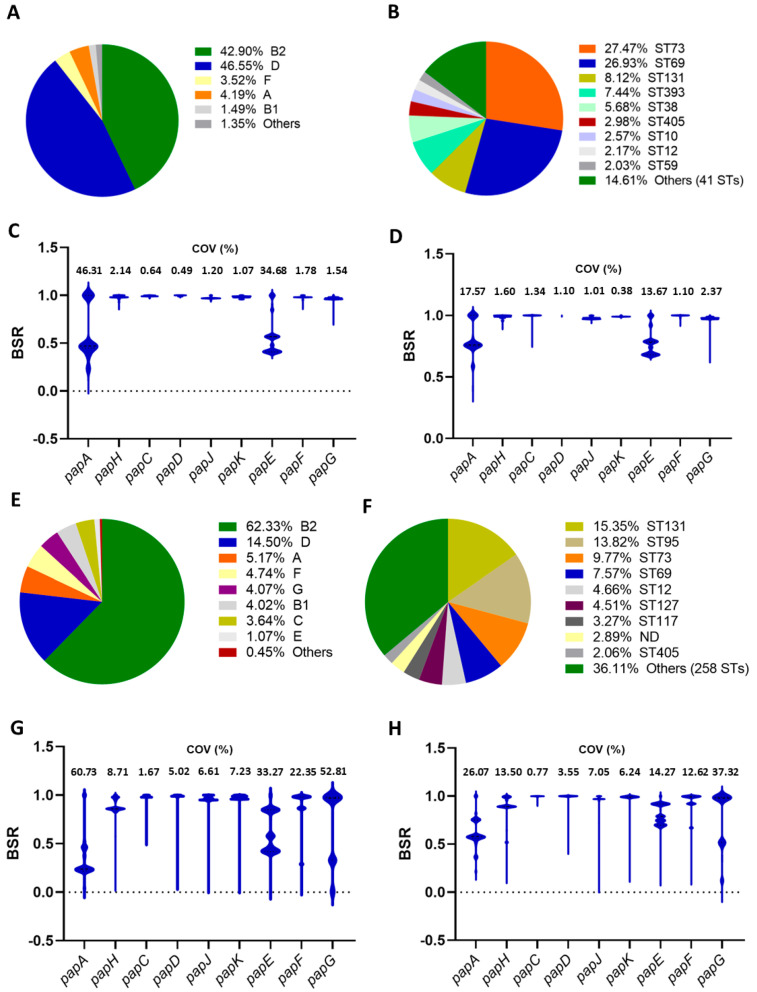
Distribution of *E. coli* genotypes among genomes positives for detecting the pap locus and screening of *pap* genes. (**A**,**B**) Distribution of phylogroups (**A**) and sequence types (**B**) among 739 genomes selected after the detection of *papAHCDJKEFG* with large-scale blast score ratio software (LS-BSR) using blastn and a cutoff of BSR ≥ 0.9. (**C**,**D**) BSR values for the screening of *pap* genes with blastn (**C**) or tblastn (**D**) among the 739 genomes. (**E**,**F**) Distribution of phylogroups (**E**) and sequence types (**F**) among 4005 genomes selected after the detection of *papC* with LS-BSR using tblastn and a cutoff of BSR ≥ 0.9. (**G**,**H**) BSR values for the screening of *pap* genes with blastn (**C**) or tblastn (**D**) among the 4005 genomes. BSR: blast score ratio, COV: coefficient of variation.

Among the 35,828 *E. coli* genomic sequences, 739 were positive, with a blast score ratio ≥ 0.9 *(BSR ≥ 0.9)* for the detection of the *papAHCDJKEFG* locus (2.1% of the isolates). Within this group, most of these strains belonged to the D (46.5%, 344 strains) and B2 (42.3%, 317 strains) phylogroups (Figure 1A). ST73 (27.5%), ST69 (27%), ST131 (8.1%), and ST393 (7.44%) were the four most common sequence types (Figure 1B). The screening of pap genes (A to G) using blastn showed a wide range of BSR values for *papA* and *papE*, with coefficients of variation of 46.31% and 34.68%, respectively, while the rest of the genes seemed to be more conserved (Figure 1C). The distribution of the BSR values for all the genes was similar after screening with the tblastn algorithm, but the coefficients of variation for *papA* and *papE* were lower (Figure 1D). In a second complementary analysis, we established the presence of the pap locus by detecting the *papC* gene, encoding the usher protein, with a BSR ≥ 0.9 after screening with the tblastn algorithm. Thus, 4005 out of 35,828 were positive (11.18%). With this criterion, most of the strains belonged to the B2 (62.33% of the records) and the D (14.50%) phylogroups (Figure 1E). ST131 (15.35%), ST95 (13.82%), ST73 (9.77%), and ST69 (7.57%) were the most common sequence types (Figure 1F). The BSR values for the screening of pap genes showed a higher variation than the results obtained in the first analysis, with *papA* and *papG* showing the highest coefficients of variation regardless of the algorithm used (blastn or tblastn, Figure 1G,H).

### 2.1. Screening of papA and papG Variants

Given that genetic variants for *papA* (F7-1, F7-2, F8, F9, F10, F11, F12, F13, F14, F15, F16, and F48) and *papG* (*papGI*, *papGII*, and *papGIII*) have been described, we explored the presence of these variants in association with the phylogroups and sequence types of the *E. coli* genomes. We considered the population of the 4005 records selected according to the criterion of BSR values ≥0.9 (tblastn) for the presence of the *papC* gene, as this allowed for the inclusion of the ST95 strains, a genotype recognized to be one of the dominants among strains causing extraintestinal infections in humans [16]. Within this universe of strains, variants for *papA* could be established in 3080 of the records, with a total sum of 4201 hits (BSR ≥ 0.9), which suggested the presence of genomes harboring more than one variant simultaneously. The most common variants were F11 (893 genomes), F10 (511 genomes), F48 (379 genomes), F16 (371 genomes), F12 (287 genomes), and F7-2 (235 genomes). Significant associations were found in twenty-one cases for pairs of a *papA* variant/phylogroup, including nine different pairs for strains belonging to the B2 phylogroup, four for F, three for D, three for C, one for B1, and one for G (Table 1). No significant associations were detected for the A and E phylogroup strains. The most common associations were F11/B2 (598 genomes), F10/B2 (447 genomes), F48/B2 (272 genomes), F16/D (265 genomes), F12/B2 (203 genomes), and F13/B2 (174 genomes) (Table 1). Forty-two cases of significant associations were found for pairs of *papA* variant/sequence types. Most cases were found in the most common *papA* variants, i.e., F7-2, F11, F10, F12, F16, and F48. Among the most frequent sequence types, the highest number of significant associations with different *papA* variants was detected for ST73 (F7-1, F7-2, F13, and F14) and ST12 (F10, F12, F13, F14, and F16). ST69 was associated with F9 and F16 variants. ST95, ST117, ST127, and ST131 strains were associated with single *papA* variants. ST95 and ST117 strains were separately associated with the F11 variant only; ST127 strains had a significant association with F48, while ST131 strains were associated with the F10 variant (Table 1). 

On the other hand, *papG* variants were also screened among the 4005 *E. coli* genome records. Variants were identified in 3459 records, and *papGII* was the most common by far, followed by *papGIII* (Table 2). Although *papGII* and *papGIII* were detected in strains belonging to all the phylogroups, they were only significantly associated with some of them. The *papGII* variant was found to be associated with phylogroups B2, D, and G, while *papGIII* was associated with phylogroup B2 only. The variant *papGI* was detected in B2 strains only (Table 2). Among the most common sequence types, ST73, ST95, and ST131 strains were found in significant association with both *papGII* and *papGIII*, ST12 with *papGI* and *papGIII*, ST69 with *papGII* only, and ST127 with *papGIII* only. 

As expected, *papGII* and *papGIII* variants were found to be significantly associated with most of the *papA* variants. In contrast, the *papGI* variant was associated with the F13 variant only (Table 2). As it is already known, some strains, such as the prototypic human UPEC strain CFT073, harbor two copies of the pap locus [29]. 

### 2.2. Genomes Harboring Two or Three papA Variants 

Our analysis identified 177 genomes (4.42% of the 4005 *papC*-positive genomes) for which 2 or 3 different *papA* variants were simultaneously detected (Table 3). Most of these genomes represent strains belonging to the B2 phylogroup (91 genomes) and to the ST12 (42 genomes), ST73 (22 genomes), and ST127 (8 genomes) sequence types (Table 3). Among them, the most common pairs identified were F11/F16, F7-2/F13, F7-2/F48, and F7-1/F14, which harbored *papGII* or the combination *papGII*/*papGIII*. In ten cases, from which seven represent ST12 strains, three different *papA* variants were simultaneously detected in combination with a single *papG* variant, *papGII*, or *papGIII*.

Among the thirty-three strains harboring two or three different *papA* variants and simultaneously one or two different *papG* variants, we could establish which pairs are contained within single pap loci only in four cases by exploring genome annotations. Thus, in three ST73 strains, the pairs F7-1/*papGII* and F14/*papGIII* (NCBI assembly code GCF_001030435.1), and F7-2/*papGII* and F13/*papGIII* (two strains, GCF_000351825.1 and GCF_025946565.1) were found. The other case was an ST12 strain, in which the pairs F9/*papGII*, and F13/*papGIII* were identified (GCF_026651165.1). In addition, to establish if strains harboring two or three different *papA* variants contained complete *papAHCDJKEFGH* loci, we explored fully sequenced genomes. Only eight records fulfilled these conditions, representing strains belonging to the B2 phylogroup only, more specifically, three sequence types and a non-typeable genome (Table 4). Complete *papAHCDJKEFGH* loci were found in all the cases, and most of them seemed to be intact, lacking disrupting elements such as premature stop codons or insertion sequences. In only one case, an F7-2 encoding locus from the record GCF_001683435.1, several frameshift mutations are informed in genome annotations, which introduce premature stop codons (Table 4). 

### 2.3. Presence of papA and papG Variants in E. coli Isolated from Humans and Animals

Given that ExPECs can be found in humans and animals, and genetic relatedness among strains has suggested the possibility of transmission between them [30], we explored host information in the genome records representing the most relevant sequence types. ST69, ST73, ST95, ST131, and ST393 were included, as well as ST12, ST117, and ST127, as they were detected among the most frequent genotypes in the selected *papC^+^* group. In seven of the eight sequence types analyzed, most genomes represent strains isolated from humans (Table 5). In fact, no animal strains were observed among ST393 strains. However, among ST12, ST69, ST73, ST95, ST127, and ST131, several records representing animal strains and harboring their main associated *papA* and/or *papG* variants were found (Table 5). The most common among these cases were the records representing ST12, ST73, ST127, and ST131 strains obtained from dogs and ST69 and ST95 strains obtained from poultry. On the other hand, ST117 strains were mostly isolated from poultry or bovines, but a minor proportion represented human strains (Table 5).

### 2.4. Screening of papA and papG Variants in a Collection of Chilean UPEC Strains

Finally, we screened *papA* and *papG* variants in a set of 17 genomes from UPEC strains isolated in Santiago, Chile, to explore if the associations found in the whole database could be detected (Table 6). Although this collection is small, representatives of the most common phylogroups and sequence types were identified. Fourteen strains belonged to phylogroup B2 and three to phylogroup D. ST73 (five genomes), ST12 (four genomes), and ST69 (three genomes) were the most frequent sequence types (Table 6). In decreasing order of frequency, *papA* variants F13, F16, F7-1, F7-2, F8, F48, F9, F11, and F14, were recognized. Variant *papGII* was detected in all the strains, with the simultaneous detection of *papGIII* in five cases. The *papGI* variant was not detected. Significant associations between *papA*/*papG* variants and the sequence types identified in NCBI Assembly Refseq genomes were detected in 16 cases within Chilean strains. Only three cases did not match with those associations: one F7-2 variant was found in an ST69 strain (92-UCH), and F13 and F11 variants were found in two different ST12 strains (175-UCH and 207-UCH) (Table 6). Three strains harboring two different *papA* variants each were identified. The pair F7-1/F48, not observed within the NCBI Assembly Refseq genome database, was detected in an ST73 strain. In addition, the pairs F9/F13 and F11/F16 were detected in two different ST12 strains, which agreed with the pairs previously found in the NCBI Assembly Refseq database (Table 6). Furthermore, loci containing *papC* and *papA*-like genes that could not be defined as any of the 12 variants screened were found in six cases. The strains 23-UCH, 151-UCH, 208-UCH, and 253-UCH harbor putative F10 homologs, 177-UCH has a putative F7-1 homolog, and 207-UCH has a putative F12 homolog. In four strains, 29-UCH, 81-UCH, 175-UCH, and 199-UCH, only one *papA* variant but two different *papG* variants were found, suggesting that other non-detected *papA* variants might be present (Table 6). In summary, most features found in the Chilean UPEC strains are consistent with those found in genomes obtained from the RefSeq Assembly database.

## 3. Discussion

P fimbria is one of the most well-known virulence factors ExPECs produce, causing infections in humans and other animals. Its role in conferring the adherence capacity has been assessed in vitro [31] and in vivo [32,33], so it has been proposed as a target for the development of anti-adherence therapies [23]. ExPECs represent a wide diversity of strains, including several phylogroups and a vast number of different sequence types [11]. This diversity is accompanied by the variability in fimbrial repertoires and the sequences of fimbrial components [19]. In this scenario, our results indicated that the gene encoding the major structural subunit PapA has the highest degree of variation compared to the rest of the genes within the *papAHCDEJKEFG* locus. This is consistent with the fact that 12 different variants had been described several years ago for PapA [23], a fact that has not been commonly reported among chaperone–usher-assembled fimbriae. Despite the vast diversity of strains, some sequence types are more frequent in extraintestinal infections, with ST69, ST73, ST95, ST131, and ST393 as the top representatives, which have been recognized as pandemic lineages [16]. This is consistent with the more common sequence types we identified within the 4005 genomes harboring *papC*, which were detected after screening the NCBI Assembly Refseq database of *E. coli* genomes with a cut-off value of BSR ≥ 0.9. In contrast, the screening of the whole *papAHCDEJKEFG* locus with the same cut-off value to establish a set of genomes representing a set of strains harboring the P fimbrial system excluded all the ST95 strains. This reflects the complexity of establishing proper cut-off values, particularly for loci containing multiple genes in draft genomes, which represent most of the genome databases today. Indeed, the availability of tools to obtain phylogroups and sequence types from multiple genomic sequences contributed to determining if our selected dataset represented *E. coli* genotypes associated with extraintestinal infections. In this case, the set of genomes we finally selected to analyze the presence of *papA* and *papG* variants included the most common genotypes as the most abundant representatives according to both categories, phylogroups, and sequence types [16,17]. A similar set could have been selected after screening other genes from the *papAHCDEJKEFG* locus. However, we selected *papC*, because the usher has previously been considered one of the most conserved components of chaperone–usher fimbrial systems and a basis for a classification scheme [34]. 

Sequence types such as ST12, ST127, ST117, and ST405 were also detected among those most common in the selected dataset for screening *papA* and *papG* variants. These sequence types have been recognized as prevalent in previous studies [17] and were detected at a higher frequency than ST393, one of the recognized pandemic lineages. Nevertheless, the number of genomes representing each one of the eight phylogroups (A to G) and the diversity of sequence types (297 sequence types) seemed to constitute a suitable universe for analyzing the distribution of *papA* and *papG* variants. Unavoidably, the number of representatives for each genotype is unequal, as the database represents the sum of genomes obtained in several studies from different geographical origins and with diverse goals. In the case of the phylogroups, the numbers were 43 genomes belonging to phylogroup E, 581 belonging to phylogroup D, and 2479 belonging to the B2 group. In the case of the sequence types, only 7 of them exceeded 100 genomes, and 39 sequence types were represented by 10 or more genomes. Among them, the most important sequence types, according to previous reports (ST10, ST12, ST69, ST73, ST88, ST95, ST117, ST127, ST131, ST393, and ST405) were represented by between 57 and 633 genomes. Regarding the association with *papA* variants, it is noteworthy that only five sequence types showed significant associations with more than one *papA* variant. ST12 and ST73 strains were found to be significantly associated with five and four different *papA* variants, respectively, while ST59, ST69, and ST457 were found to be associated with two variants each. Even the most abundant sequence types, ST131 and ST95, were significantly associated with only one *papA* variant. Therefore, specific features and/or separate evolutionary events, particularly for ST12 and ST73 strains, might determine that those lineages harbor two or more *papA* variants. Accordingly, ST73 and ST12 were the only ones among the eleven more frequent sequence types that were found significantly associated with two different *papG* variants. 

Furthermore, in the cases in which two or three different *papA* and two different *papG* variants were simultaneously detected, the exploration of complete genomes confirmed the presence of complete *papAHCDJKEFG* loci. This was the already-known case of the UPEC CFT073 strain [29]. In addition, our results are consistent with the results of a previous report in which the location of the P fimbria locus was analyzed [35]. It is known that the locus might be contained in pathogenicity islands (PAIs) located downstream of tRNA genes or within *ula* or *gln* operons. Thus, the location of *pap*-containing PAIs was found to differ among *E. coli* genotypes (phylogroups and sequence types). Also, for the strains harboring two or three copies, *pap*-containing PAIs were found to be inserted in two or three of the hot spots [35]. This observation suggests that P fimbria production could be directed from both loci and coexist at the surface of a single bacterium. In fact, different combinations of *papA* and *papG* variants could be generated if the minimum number of genes is functional. One of the complete genomes that we analyzed, corresponding to *E. coli* BH100 substr. MG2014, has frameshifting mutations on *papC* and *papGIII* genes in one of its two *papAHCDJKEFG* loci, which introduce premature stop codons. Perhaps, for other *E. coli* genomes that were not explored in this work (NCBI Assembly non-Refseq records), non-functional genes may also be present. If transcription and translation of the intact genes occur, proteins derived from any locus could complement the formation of functional P fimbriae. Further research will be required to determine the presence rate of intact and non-functional genes within *papAHCDJKEFG* loci as well as the regulation of their expression.

The presence of *E. coli* strains harboring similar features to those found in human ExPECs, in poultry, in farm animals, and also in accompanying pets such as dogs and cats have raised the hypotheses of a common origin for these strains and also the possibility of zoonotic transmissions in cases of extraintestinal infections in humans [30,36,37]. Consistent with these data, several genomes from strains obtained from human and non-human hosts, belonging to the B2 and D phylogroups and to the ST12, ST69, ST73, ST95, ST127, and ST131 sequence types, were found in the NCBI Assembly RefSeq database. By far, most of the strains that had host information were obtained from humans. However, we detected genomes of animal *E. coli* strains belonging to these genotypes, which harbor the same *papA* and *papG* variants as those isolated from humans. This is consistent with the hypothesis of a common origin and/or transmission among humans and animals and between animals [30]. Noteworthy was the detection of genomes harboring F11 and *papGII* in ST95 strains from humans and poultry and ST12 strains obtained from dogs harboring F10, F12, or F13 and *papGI* or *papGII*. An opposite distribution was observed for ST117 genomes, which mainly represent strains isolated from poultry and bovines but include some representing human strains. Given that the contact between humans and poultry, humans and farm animals, or humans and dogs is common and can be sustained in time, the transmission of ExPECs could be feasible. On the other hand, as it has been suggested, ST73 strains harboring F14 and *papGII* isolated from orcas likely represent the contamination of the environment by animal strains [38]. 

As expected, the most common ExPEC genotypes were also found in UPEC strains isolated in Chile. Although this collection is small, most of their *papA* and *papG* variants agreed with significant associations detected in the analysis of the genomes contained in the NCBI Assembly Refseq database. Specifically, those associations were found in eight cases distributed along 16 strains. In fact, some of them were found in two cases or more. The profile ST12/F13 was found in four cases, and profiles ST14/F8, ST69/F16, and ST73/F7-1, in two cases each. Only three exceptions for *papA* variants were noticed, F7-2 in an ST69 strain and F11 in two different ST12 strains. In addition, the variant *papGII* was detected in ST12 strains, an association that was not observed in the database. Another coincidence is that the strains harboring two different *papA* variants were ST12 or ST73 strains. Furthermore, in three ST12 strains harboring the F13 variant, two different *papG* variants were detected, suggesting the presence of two pap loci. However, the sequencing data did not allow for the detection of another *papA* variant. Overall, this study represents the first characterization of UPEC genomes obtained in Chile. As the databases represent a sum of genomes of strains obtained from diverse geographic sites, it is expected to find variability when local populations are analyzed. Further studies will report other features of these strains regarding antimicrobial resistance and virulence factor profiles. 

In summary, this study showed a general picture of the presence of *papA* and *papG* variants among *E. coli* phylogroups and sequence types based on the genomes contained in the NCBI Assembly Refseq database and the genomes of ExPECs isolated in Chile. We hope this descriptive study and the associations found here serve as a general guide for future epidemiological studies to follow up on the distribution of the *pap* locus among *E. coli* strains. Our findings can be valuable for characterizing the pandemic ExPEC lineages ST69, ST73, ST95, ST131, and ST393, belonging to phylogroups B2 and D, but also for other potential emerging lineages, which could incorporate the *pap* locus by horizontal transference. With this scenario, we highlight the relevance and versatility of ST12 strains, found as a common lineage associated with infection and humans but also isolated from canines, that can harbor two or three *papAHCDJKEFG* loci, including several *papA* variants. The hypothesis of a functional adaptative advantage conferred by the most common *papA* and *papG* variants has been previously raised, and it can certainly be proposed based on our results. Thus, P fimbriae produced by the most common lineages, composed of the PapA variants F11, F10, F48, F16, F12, or F7-2 and *papGII*-derived tip subunits, would be more efficient in their role of conferring colonization capacities compared to structures harboring the less common variants. Further research could test these hypotheses. Indeed, having a landscape of the current distribution of *papA* and *papG* variants might also help to establish directions in the efforts to advance the knowledge of the P fimbria-mediated ExPEC adherence/colonization mechanism and to develop P fimbria-based anti-virulence therapies.

## 4. Materials and Methods

*Screening of pap genes*: A total of 35.828 *E. coli* genomes were obtained from the Assembly Refseq database available in the National Center for Biotechnology Information (NCBI) of the United States of America (https://www.ncbi.nlm.nih.gov/genbank/, accessed on 8 January 2024) [39]. The presence of the *pap* locus, encoding the P fimbria, was established in two ways. First, *papAHCDJKEFG*, as a single DNA sequence, was screened using large-scale blast score ratio (LS-BSR) with the blastn algorithm (available at https://github.com/jasonsahl/LS-BSR, accessed on 8 January 2024) [40]. Genomic sequences with BSR ≥ 0.9 were considered as positive. The sequences obtained from the databases for screening purposes are described in Table S1. Alternatively, the presence of the *papC* gene (BSR ≥ 0.9), encoding the usher protein, was used as indicative of the presence of the *papAHCDJKEFG* locus. The genes *papA*, *papH*, *papC*, *papD*, *papJ*, *papK*, *papE*, *papF*, and *papG* were also screened individually using LS-BSR with the blastn and tblastn algorithms [40]. Furthermore, genes encoding the *papA* variants F7-1, F7-2, F8, F9, F10, F11, F12, F13, F14, F15, F16, and F48 and *papG* variants *papGI*, *papGII*, and *papGIII* were screened with LS-BSR using tblastn. Records with BSR ≥ 0.9 were considered positive for a variant, except for F12, F15, and F16, which are highly similar among them. Therefore, BSR ≥ 0.96, BSR ≥ 0.99, and BSR ≥ 0.97, respectively, were considered as cut-off values in those cases. The phylogroup was assigned using EzClermont v0.6.3, and the sequence type was determined using mlst v2.18, according to the Achtman’s scheme [41,42,43]. Graphics, distribution, and association analyses were performed using GraphPad Prism v9 software. Associations were established according to Fisher’s exact test and the Chi-square test, and they were reported when the number of genomes displaying a particular feature was higher or equal to ten (with the only exception of the presence of F8 in association with the *papGI* variant in Table 2) and when the odds ratio >1.0.

*Sequencing of Chilean UPEC Strains*: Eleven UPEC strains, isolated from urosepsis cases, were taken from a collection stored at the Programa de Microbiología y Micología, Instituto de Ciencias Biomédicas, Facultad de Medicina, Universidad de Chile. All of these strains were isolated from blood cultures in a previous study carried out in three hospitals located in Santiago, Chile: Hospital Dr. Félix Bulnes, Hospital Dr. Exequiel González-Cortés, and Hospital Padre Hurtado [44]. The use of these strains was authorized by JMI Laboratories, the owner of the collection, and also by the Ethics Committee of the Facultad de Medicina, Universidad de Chile (Ethics Approval Document N° 003, issued on 4 May 2023). In addition, six UPEC strains were obtained from routine urine cultures performed at the Hospital Clínico de la Universidad de Chile. The use of these strains was authorized by the Ethics and Scientific Committee of the Hospital Clínico de la Universidad de Chile and the Ethics Committee of the Facultad de Medicina, Universidad de Chile (Ethics Approval Document N° 01, issued on 18 January 2018). Details of the strains are shown in Table 6. The strains were cultured overnight at 37 °C in lysogeny broth (LB, Lennox formula), and genomic DNA was purified using a commercial kit, according to the manufacturer’s instructions (Wizard Genomic DNA Purification kit, Promega, Madison, WI, USA). The integrity of the DNA was checked using electrophoresis in 1% agarose gel and ethidium bromide staining. Sequencing was performed at MicrobesNG (Birmingham, UK) using the Illumina MiSeq platform (Illumina Inc., San Diego, CA, USA). The details of the sequences obtained are shown in Table S2. The draft genomes obtained after assembly with SPAdes v3.14 [45] were provided and checked by using QUAST v5.0.2 [46] and CheckM v1.2.2 [47]. The species identity was corroborated using “Identify Species” (available at https://pubmlst.org/species-id, accessed on 8 January 2024) [48]. The *E. coli* phylogroup, sequence types, and the presence of genes encoding P fimbria, including *papA* and *papG* variants, were determined as indicated above for the genomic sequences recovered from databases.

## Figures and Tables

**Table 1:** Distribution of *papA* variants among *papC^+^ E. coli* genomes from NCBI Assembly Refseq database.

*papA* Variant	N° of Positive Genomes	Distribution among Phylogroups (Phylogroup: n° Genomes)	Significant Association with Sequence Types: n° Genomes (n° Genomes per Phylogroup)
F7-1	62	B2: 57 *** D: 2 F: 3	ST73: 25 *** (25 B2) ST144: 19 *** (19 B2)
F7-2	235	B2: 170 * D: 61 *** A: 1 B1: 1 G:1 U:1	ST73: 136 *** (136 B2) ST38: 37 *** (35 D, 1 G, 1U) ST405: 10 * (10 D)
F8	59	B2: 25 F: 21 *** A: 9 D: 3 G: 1	ST14: 23 *** (23 B2) ST59: 20 *** (19 F, 1 G)
F9	85	B2: 37 D: 24 ** F: 20 *** A: 2 G: 2	ST69: 19 *** (19 D) ST59: 18 *** (17 F, 1 G)
F10	511	B2: 447 *** F: 38 ** A: 9 D: 7 B1: 4 C: 4 Cryptic: 1 U: 1	ST131: 202 *** (202 B2) ST12: 107 *** (107 B2) ST62: 28 *** (28 F) ST625: 10 *** (10 B2)
F11	893	B2: 598 ** G: 86 *** C: 57 *** B1: 40 F: 35 A: 33 D: 22 E: 14 U: 8	ST95: 477 *** (475 B2, 2 U) ST117: 66 *** (66 G) ST88: 39 *** (39 C) ST457: 22 *** (22 F) ST421: 21 *** (21 B2) ST58: 18 *** (18 B1) ST5935: 17 *** (17 G)
F12	287	B2: 203 * F: 22 * A: 21 B1: 17 D: 13 G: 5 C: 3 E: 2 Cryptic: 1	ST12: 31 *** (31 B2) ST141: 29 *** (29 B2) ST457: 13 *** (13 F) ST961: 11 *** (10 B2, 1 cryptic) ST83: 10 *** (10 B2) ST706: 10 *** (10 B2) ST773: 10 *** (10 A)
F13	231	B2: 174 *** C: 16 ** A: 9 B1: 8 E: 8 F: 8 D: 5 U: 3	ST73: 35 * (34 B2, 1 U) ST12: 24 ** (24 B2) ST372: 22 *** (22 B2) ST410: 16 *** (15 C, 1 U) ST998: 11 *** (11 B2)
F14	137	B2: 119 *** D: 7 A: 4 C: 3 B1: 2 F: 1 G: 1	ST73: 67 *** (67 B2) ST12: 14 * (14 B2)
F15	15	B2: 14 * A: 1	ND
F16	371	D: 265 *** B2: 75 A: 23 F: 5 B1: 2 G: 1	ST69: 182 *** (181 D, 1 G) ST12: 49 *** (49 B2) ST393: 57 *** (57 D) ST10: 16 ** (16 A) ST827: 10 *** (10 B2)
F48	379	B2: 272 * C: 35 *** B1: 32 *** D: 17 A: 15 F: 15 E: 2 G: 1 U: 1	ST127: 170 *** (169 B2, 1 U) ST648: 13 ** (13 F) ST23: 12 *** (12 C)

U: unassigned phylogroup. ND: not detected. * *p* < 0.05, ** *p* < 0.001, and *** *p* < 0.0001, according to Fisher’s exact test and the Chi-square test.

**Table 2:** Distribution of *papG* variants among *papC^+^ E. coli* genomes from NCBI Assembly Refseq database.

*papG* Variant	N° of Positive Genomes	Distribution among Phylogroups (Phylogroup: n° Genomes)	Significant Association with Sequence Types (ST: n° Genomes per Phylogroup)	Significant Association with *papA* Variants (n° Genomes per ST)
*papGI*	26	B2: 26 ***	ST12: 14 *** (14 B2)	F13: 8 ***
*papGII*	2569	B2: 1632 * D: 474 *** A: 124 F: 108 G: 90 * B1: 69 C: 34 E: 26 U: 12	ST131: 584 *** (584 B2) ST95: 480 *** (478 B2, 2U) ST73: 295 *** (294 B2, 1 U) ST69: 246 *** (245 D, 1 G) ST405: 83 *** (82 D, 1 G) ST393: 57 *** (57 D) ST38: 48 *** (46 D, 1 G, 1 U) ST62: 35 *** (35 F) ST144: 32 *** (32 B2) ST14: 28 *** (28 B2) ST421: 27 *** (27 B2) ST617: 11 * (A)	F11: 623 *** F16: 346 *** F7-2: 223 *** F9: 76 *** F7-1: 59 *** F8: 24 *
*papGIII*	942	B2: 787 *** B1: 45 F: 42 C: 40 A: 12 D: 6 G: 4 U: 3 E: 2 Cryptic: 1	ST127: 153 *** (152 B2, 1 U) ST73: 138 *** (138 B2) ST12: 122 *** (122 B2) ST141: 39 *** (39 B2) ST372: 37 *** (37 B2) ST457: 34 *** (34 F) ST410: 19 *** (18 C, 1 U) ST101: 18 *** (18 B1) ST625: 13 *** (13 B2) ST998: 11 *** (11 B2) ST83: 10 *** (10 B2) ST961: 10 *** (9 B2, 1 cryptic)	F48: 247 *** F10: 198 *** F12: 192 ** F13: 155 *** F14: 90 ***

U: unassigned phylogroup. * *p* < 0.05, ** *p* < 0.001, and *** *p* < 0.0001, according to Fisher’s exact test and the Chi-square test.

**Table 3:** Features of *E. coli papC*^+^ genomes positives for two or three *papA* variants.

*papA* Variants	N° of Genomes	Phylogroups (Phylogroup: n° Genomes)	Sequence Types (ST: n° Genomes)	*papG* Variants (Variant: n° Genomes)
F7-1/F7-2	3	B2: 3	ST73: 3	*papGII*: 3
F7-1/F10	2	B2: 2	ST144: 2	*papGII:* 2
F7-1/F12	1	B2: 1	ST73: 1	*papGIII*: 1
F7-1/F14	8	B2: 8	ST73: 8	*papGII*/*papGIII*: 8
F7-1/F16	1	B2: 1	ST12: 1	*papGII*: 1
F7-2/F10	4	B2: 3	ST6355: 3	*papGII: 2*
		D: 4	ST8767: 1	*papGII: 1*
F7-2/F13	11	B2: 11	ST73: 10	*papGII:* 2 *papGII*/*papGIII*: 8
			ST131: 1	*papGII*: 1
F7-2/F48	8	B2: 8	ST127: 7	*papGII*: 8
			ST8312: 1	*papGIII*: 1
F8/F9	15	F: 14	ST59: 13	*papGII*: 13
			ST6199: 1	*papGII*: 1
		G: 1	ST59: 1	*papGII*: 1
F8/F14	1	F: 1	ST59: 1	*papGII*: 1
F9/F11	1	B2: 1	ST12: 1	*papGII*: 1
F9/F12	1	B2: 1	ST141: 1	ND: 1
F9/F13	5	B2: 5	ST12: 5	*papGII*: 1 *papGII*/*papGIII*: 4
F9/F15	1	B2: 1	ST8118: 1	*papGII*: 1
F11/F13	2	B2: 2	ST12: 2	*papGII*/*papGIII*: 2
F10/F11	1	B2:1	ST12: 1	*papGII*: 1
F10/F12	33	B2: 32	ST12: 25	*papGIII*: 24 ND: 1
			ST625: 1	*papGI/papGIII*: 1
			ST961: 3	*papGIII*: 2 ND: 1
			ST2604: 3	*papGIII*: 3
		Cryptic: 1	ST961: 1	*papGIII*: 1
F10/F13	1	B2: 1	ST131: 1	*papGIII*: 1
F10/F14	1	B2: 1	ST12: 1	*papGIII*: 1
F10/F16	15	B2: 15	ST12: 15	*papGII*: 15
F11/F13	2	B2: 2	ST12: 2	*papGI/papGIII*: 2
F11/F14	2	B2: 1	ST12: 1	*papGII*/*papGIII*: 1
		C: 1	ST88: 1	*papGIII*: 1
F11/F16	25	B2: 25	ST12: 25	*papGII*: 24 ND: 1
F11/F48	2	A: 1	ST10: 1	*papGII*: 1
		B2: 1	ST131: 1	*papGIII*: 1
F12/F14	2	B2: 2	ST12: 1	*papGIII*: 1
			ST372: 1	*papGII*/*papGIII*: 1
F12/F48	6	B2: 6	ST144: 6	*papGII*: 6
F13/F14	5	B2: 5	ST12: 3	*papGI*/*papGIII*: 1 *papGII*/*papGIII*: 1 ND: 1
			ST599: 2	*papGI*/*papGIII*: 2
F13/F48	2	B2: 2	ST555: 2	*papGII*/*papGIII*: 2
F14/F16	4	B2: 3	ST12: 3	*papGII*: 3
		D: 1	ST69: 1	*papGII*: 1
F15/F16	2	B2: 2	ST827: 2	*papGIII*: 2
F7-1/F9/F15	1	B2: 1	ST703: 1	ND: 1
F7-1/F15/F16	1	B2: 1	ST73: 1	*papGIII: 1*
F9/F10/F16	1	B2: 1	ST12: 1	*papGII*: 1
F9/F11/F16	1	B2: 1	ST12: 1	*papGII*: 1
F10/F11/F16	3	B2: 3	ST12: 3	*papGII*: 3
F10/F14/F16	1	B2: 1	ST12: 1	ND
F13/F14/F16	2	B2: 2	ST12: 1	ND
			ND: 1	*papGII*: 1

ND: not detected.

**Table 4:** Full *E. coli* genomes harboring two or three *papAHCDEJKEFG* loci and their associated *papA* and *papG* variants.

Strain (NCBI Assembly Code)	Phylogroup/ST	1st *papA*/*papG* Pair (*pap* Locus Coordinates)	2nd *papA*/*papG* Pair (*pap* Locus Coordinates)	3rd *papA*/*papG* Pair (*pap* Locus Coordinates)
*E. coli* GN02350 (GCF_026651165.1)	B2/ST12	F13/*papGII* (c1,566,503–-1,558,580)	F9/*papGIII* (c2,122,156–2,114,207)	-
*E. coli* C 691-04A GCF_025946565.1	B2/ST73	F7-2/*papGIII* (899,304–907,248)	F13/*papGIII* (c4,560,907–4,552,986)	-
*E. coli* CFT073 GCF_014262945.1	B2/ST73	F7-2/*papGII* (c3,448,359–3,440,421)	F7-1/*papGII* (c4,959,718–4,951,783)	-
*E. coli* BH100N substr. MG2017 GCF_002900305.1	B2/ST127	F48/*papGIII* (c3,287,322–3,279,405)	F7-2/*papGIII* (c4,881,632–4,873,708)	-
*E. coli* BH100 substr. MG2014 GCF_002763515.2	B2/ST127	F48/*papGIII* (c3,214,562–3,206,645)	F7-2/*papGIII* (c4,824,548–4,816,627) ^FS^	-
*E. coli* K-15KW01 GCF_001683435.1	B2/ST127	F7-2/*papGIII* (c1,676,021–1,669,148)	F48/*papGIII* (c3,310,504–3,302,587)	-
*E. coli* EC5654 GCF_022919035.1	B2/ST12	F16/*papGII* (878,908–886,816)	F10/*papGII* (4,458,416–4,466,350)	F11/*papGII* (c4,638,209–4,630,290)
*E. coli* strain UPEC132 GCF_007833875.1	B2/ND	F14/*papGII* (79,054–86,999)	F16/*papGII* (934,430–942,336)	F13/*papGII* (c4,629,322–4,621,398)

^FS^: frameshifting mutations are reported within *papC* and *papGIII* genes.

**Table 5:** Hosts of ST131, ST95, ST73, ST12, ST127, ST117, and ST393 *papC^+^ E. coli* genomes harboring their significantly associated *papA* and *papG* variants.

Sequence Type	*papA*/*papG* Variant	N° Genomes	N° Genomes with Host Information	N° Genomes per Host
ST131	All	633	531	Human: 505
				Dog: 16
				Others: 10 (6 types)
	F14	10	9	Human: 8
				Porcine: 1
	F48	43	39	Human: 38
				Dog: 1
	*papGII*	584	487	Human: 464
				Dog: 14
				Others: 9 (6 types)
	*papGIII*	41	37	Human: 34
				Dog: 2
				Porcine: 1
ST95	All	570	513	Human: 427
				Poultry: 69
				Others: 16 (9 types)
	F11	477	434	Human: 350
				Poultry: 69
				Others: 15 (8 types)
	*papGII*	480	436	Human: 351
				Poultry: 69
				Others: 16 (9 types)
	*papGIII*	85	72	Human: 71
				Non-human primate: 1
ST73	All	403	305	Human: 278
				Dog: 8
				Feline: 3
				Others: 16 (10 types)
	F7-1	29	25	Human: 24
				Dog: 1
	F7-2	135	62	Human: 61
				Porcine: 1
	F13	35	31	Human: 28
				Non-human primate: 3
	F14	67	65	Human: 61
				Orca: 4
	F48	15	13	Human: 9
				Dog: 3
				Common polecat: 1
	*papGII*	295	208	Human: 199
				Orca: 4
				Others: 5 (4 types)
	*papGIII*	138	124	Human: 110
				Dog: 7
				Others: 7 (5 types)
ST69	All	312	261	Human: 241
				Poultry: 9
				Others: 6 (4 types)
	F9	19	12	Human: 12
	F16	182	149	Human: 147
				Others: 2 (2 types)
	*papGII*	246	205	Human: 191
				Poultry: 8
				Others: 6 (4 types)
ST12	All	192	173	Human: 140
				Dog: 16
				Others: 17 (13 types)
	F10	107	92	Human: 69
				Dog: 9
				Others: 14 (12 types)
	F12	31	29	Human: 20
				Dog: 4
				Others: 5 (4 types)
	F13	24	23	Human: 19
				Dog: 4
	F14	14	13	Human: 11
				Others: 2 (2 types)
	F16	49	45	Human: 45
	*papGI*	14	13	Human: 6
				Dog: 4
				Others: 3 (3 types)
	*papGIII*	122	108	Human: 77
				Dog: 16
				Others: 15 (11 types)
ST127	All	186	168	Human: 144
				Dog: 7
				Others: 17 (13 types)
	F48	170	153	Human: 136
				Dog: 4
				Others: 13 (9 types)
	*papGIII*	153	136	Human: 118
				Dog: 5
				Others: 13 (10 types)
ST117	All	135	116	Poultry: 69
				Bovine: 21
				Human: 9
				Others: 18 (6 types)
	F11	66	61	Poultry: 41
				Bovine: 8
				Human: 5
				Others: 7 (3 types)
ST393	All	57	53	Human: 53
	F16	57	53	Human: 53
	*papGII*	57	53	Human: 53

**Table 6:** Main features of UPEC strains isolated in Chile.

Strain	Diagnosis	Year of Isolation	Phylogroup	Sequence Type	*papA* Variant	*papG* Variant
23-UCH	Urosepsis	2011	B2	ST14	F8	*papGII*
29-UCH	Urosepsis	2011	B2	ST12	F13	*papGII*/*papGIII*
81-UCH	Urosepsis	2009	B2	ST12	F13	*papGII*/*papGIII*
92-UCH	Urosepsis	2009	D	ST69	F7-2	*papGII*
104-UCH	Urosepsis	2009	D	ST69	F16	*papGII*
112-UCH	Urosepsis	2009	D	ST69	F16	*papGII*
150-UCH	Urosepsis	2008	B2	ST131	F48	*papGII*
151-UCH	Urosepsis	2008	B2	ST73	F7-1/F48	*papGII*
175-UCH	Urosepsis	2008	B2	ST12	F13	*papGII*/*papGIII*
176-UCH	Urosepsis	2008	B2	ST73	F7-2	*papGII*
177-UCH	Urosepsis	2008	B2	ST12	F9/F13	*papGII*/*papGIII*
197-UCH	UTI	2017	B2	ST73	F7-1	*papGII*
199-UCH	UTI	2017	B2	ST73	F14	*papGII*/*papGIII*
207-UCH	UTI	2017	B2	ST12	F11/F16	*papGII*
208-UCH	UTI	2017	B2	ST73	F13	*papGII*
235-UCH	UTI	2017	B2	ST131	ND	*papGII*
253-UCH	UTI	2017	B2	ST14	F8	*papGII*

UTI: urinary tract infection. ND: not detected.

## Data Availability

Genomic sequences obtained in this work were deposited in Genbank (National Center for Biotechnology Information, Bethesda, MD, USA) under the BioProject code PRJNA930773.

## Supplementary Materials

The following supporting information can be downloaded at: https://www.mdpi.com/article/10.3390/ijms25126657/s1.
