# Supplementary material for: Distribution of papA and papG Variants among Escherichia coli Genotypes: Association with Major Extraintestinal Pathogenic Lineages
Source: Int J Mol Sci. 2024 Jun 17;25(12):6657. doi: 10.3390/ijms25126657 (PMC11203468; doi:10.3390/ijms25126657)
Supplement: Supplementary file 1 [file ijms-25-06657-s001.zip › Table_S1-accession_codes.pdf]

Table S1. Accession codes of sequences used in this study for screening purposes

Locus / gene / variant	NCBI Nucleotide Accession code	Position
papAHCDJKEFG	NZ_CP051263.1	c 3,440,421 – 3,448,359
papA	NZ_CP051263.1	c 3,447,793 – 3,448,359
papH	NZ_CP051263.1	c 3,447,136 – 3,447,723
papC	NZ_CP051263.1	c 3,444,567 – 3,447,077
papD	NZ_CP051263.1	c 3,443,762 – 3,444,481
papJ	NZ_CP051263.1	c 3,443,144 – 3,443,725
papK	NZ_CP051263.1	c 3,442,598 – 3,443,134
papE	NZ_CP051263.1	c 3,442,050 – 3,442,571
papF	NZ_CP051263.1	c 3,441,475 – 3,441,975
papG	NZ_CP051263.1	c 3,440,421 – 3,441,431
F7-1	X02921.1	184 - 747
F7-2	M12861.1	197 - 763
F8	Y08931.1	1 - 564
F9	M68059.1	37 - 612
F10	Y08927.1	1 - 531
F11	L07420.2	1,176 – 1,724
F12	CP122451.1	4,515,828 – 4,516,364
F13	X61239.1	1,763 – 2,320
F14	CP141080.1	4,722,281 – 4,722,823
F15	Y08929.1	1 - 507
F16	Y08930.1	1 - 504
F48	AF234626.1	1 - 519
papGI	X61239.1	8,679 – 9,686
papGII	M20181.1	1,222 – 2,235
papGIII	X61238.1	1,223 – 2,230

c: reverse complementary strand
