# Supplementary material for: Distribution of papA and papG Variants among Escherichia coli Genotypes: Association with Major Extraintestinal Pathogenic Lineages
Source: Int J Mol Sci. 2024 Jun 17;25(12):6657. doi: 10.3390/ijms25126657 (PMC11203468; doi:10.3390/ijms25126657)
Supplement: Supplementary file 1 [file ijms-25-06657-s001.zip › Table_S2-genome_metrics.pdf]

Table S2. Genome profiles of UPEC strains included in this study

Strain	Sequence type	Genome size (bp)	N50 (bp)	Completeness (%)	Contamination (%)	Genbank Nucleotide Accession Code
23-UCH	ST14	5,396,067	358,842	99.65	0.08	JAQQRC000000000
29-UCH	ST12	5,296,566	370,552	99.96	0.08	JAQQRB000000000
81-UCH	ST12	5,298,361	362,179	99.96	0.08	JAQQQT000000000
92-UCH	ST69	5,415,222	188,996	99.96	0.13	JAQQQS000000000
104-UCH	ST69	5,527,817	134,749	99.96	0.67	JAQQQR000000000
112-UCH	ST69	5,472,088	188,501	99.96	0.13	JAQQQQ000000000
150-UCH	ST131	5,341,975	251,876	99.96	0.72	JAQQQN000000000
151-UCH	ST73	5,341,938	368,799	99.96	0.08	JAQQQM000000000
175-UCH	ST12	5,087,106	298,386	99.96	0.08	JAQQQL000000000
176-UCH	ST73	5,138,739	307,350	99.96	0.08	JAQQQK000000000
177-UCH	ST12	5,243,990	373,763	99.96	0.08	JAQQQJ000000000
197-UCH	ST73	5,291,959	465,008	99.96	0.08	JAQQQI000000000
199-UCH	ST73	5,142,620	247,662	99.96	0.08	JAQQQG000000000
207-UCH	ST12	5,247,993	236,835	99.96	0.08	JAQQQF000000000
208-UCH	ST73	5,228,567	425,059	99.34	0.08	JAQQQE000000000
235-UCH	ST131	5,748,677	256,891	99.96	0.42	JAQQQD000000000
253-UCH	ST14	5,456,080	240,885	99.65	0.08	JAQQQB000000000
